# Retreatment of Classical Tic Douloureux With Stereotactic Radiosurgery: A Scoping Review

**DOI:** 10.7759/cureus.45468

**Published:** 2023-09-18

**Authors:** Mahima Goel, Nitin U Patil, Romalpreet Singh, Janice John, Bipin R Upadhyay

**Affiliations:** 1 Department of Oral and Maxillofacial Surgery, Pacific Dental College and Research Centre, Udaipur, IND; 2 Department of Oral and Maxillofacial Surgery, Jawahar Medical Foundation's Annasaheb Chudaman Patil Memorial Dental College, Dhule, IND; 3 Department of Conservative Dentistry and Endodontics, Desh Bhagat Dental College, Malout, IND; 4 Department of Dentistry, K. J. Somaiya Medical College, Mumbai, IND

**Keywords:** facial numbness, tic douloureux, pain, stereotactic radiosurgery, scoping review

## Abstract

Stereotactic radiosurgery (SRS), also known as gamma knife surgery (GKS), is a noninvasive procedure for treating tic douloureux (TD) or trigeminal neuralgia (TN). Due to a lack of sufficient evidence regarding the indication of SRS for the treatment of recurrent TD, the present scoping review was conducted to assess the effectiveness of repeated SRS procedures for managing recurrent TD. The literature search was performed from January 2012 to December 2022 on the PubMed, Scopus, and Web of Science databases. Of the 215 initial results obtained, 10 articles were finally selected for the review. Three studies used the SRS procedure for the third time in patients with recurrent TD. All studies were retrospective, with a mean maximal dose of 70-90 Gy and a cumulative dose of 120-180 Gy for two SRS treatments and 150-270 Gy for three SRS treatments. The target zone for irradiation was the retrogasserian zone (RGZ). Repeat SRS procedures led to pain relief in 80-90% of patients within one to four months and excellent pain relief in 50-90% of patients. Pain recurrence was noticed after one year in 20-40% of patients. Postoperative complications, such as trigeminal nerve deficits, facial numbness, and mild corneal dryness, were noted in the studies. The review concluded that repeat SRS is an effective and relatively safe procedure for pain management in patients with recurrent TD.

## Introduction and background

Tic douloureux (TD), also referred to as trigeminal neuralgia (TN), is a condition characterized by chronic pain disorder of acute onset and severity that specifically targets the facial region. This condition fundamentally involves the trigeminal nerve, also known as the fifth cranial nerve, which is responsible for both sensation and neural communication throughout various regions of the cranium and face [1]. The diagnosis of this condition is primarily established based on clinical grounds due to the manifestation of typical symptoms and signs, including paroxysmal attacks that occur abruptly and last for a duration ranging from seconds to two minutes, intervals of spontaneous remission, the presence of "trigger zones," the absence of any objective neurological deficit, the lack of any other discernible causes of facial pain, and the most common distribution along the second or third division of the trigeminal nerve [2]. The primary pathophysiological mechanism is the demyelination of sensory trigeminal afferents, specifically in the root entry zone. Demyelination can be attributed to neurovascular conflict, which subsequently causes morphological modifications, including compression of the trigeminal root [2].

TN is classified into two types: classical trigeminal neuralgia (CTN), associated with an unknown etiology and often treated with neurovascular decompression, and secondary trigeminal neuralgia (STN), associated with multiple sclerosis [1]. The first-line treatment for TN is drug therapy involving carbamazepine, and the second-line treatment involves various surgical procedures, such as microvascular decompression (MVD), gamma knife radiosurgery (GKS), percutaneous balloon compression (PBC), glycerol rhizotomy (GR), and radiofrequency thermocoagulation procedures (RTP) [3].

TN has a high recurrence rate of 6-41%. Previous studies demonstrated that the retreatment rate was 20% following MVD, 6% following GKS, and 57% following percutaneous radiofrequency rhizotomy [4]. Patients who continue to experience residual or recurrent pain may require additional therapy, including radiosurgery or alternative modalities. Gamma knife surgery is employed to manage recurring facial pain or pain that remains inadequately controlled post-initial intervention [5].

GKS, also known as stereotactic radiosurgery (SRS), is a commonly used treatment modality for refractory facial pain associated with TN that involves the trigeminal nerve. The concept of radiosurgery was first presented by Lars Leksell in 1951 [6]. SRS uses multiple focused beams from a cobalt-60 source. Repeat SRS is an efficacious treatment with a low incidence of complications in patients with recurrent or refractory TN [5]. Since 1996, there has been a continuous increase in literature advocating the safety and efficacy of SRS for the treatment of recurrent TD. However, there is a paucity of studies describing the efficacy of repeat radiosurgery. Moreover, there is a lack of clear guidelines pertaining to the indication or technique for repeat radiosurgery, including patient selection criteria for retreatment and dosing [7]. Some authors recommend the use of low doses in the second SRS [8], whereas others have recommended higher doses to be effective for the treatment of recurrent TD [9].

To date, only one systematic review was conducted in 2014 to evaluate the effectiveness of the repeat SRS procedure in the treatment of recurrent TD [7]. In light of various controversies surrounding the role of SRS in the management of recurrent TD and the lack of sufficient current literature on this topic, the present scoping was conducted to answer the main question: “Is SRS an efficient procedure for the treatment of recurrent TD or not?” The secondary questions were as follows: RQ1: Are the results different between males and females? RQ2: What are the demographic characteristics (age, sex, ethnicity, and type of study) of the studies? RQ3: What was the duration of the follow-up period in the patients used in this study? RQ4: What is the recurrence rate in patients after the initial procedure? RQ5: What are the optimal doses used for initial and recurrent treatment? RQ6: What is the optimal target? RQ7: What were the outcomes of the second procedure in recurrent cases?

## Review

Methods

The Preferred Reporting Items for Systematic Reviews and Meta-Analyses extension for Scoping Reviews (PRISMA-ScR) guidelines and Joanna Briggs Institute (JBI) protocol were followed in this review to provide a clear and transparent reporting of the outcomes. Ethical committee approval was not required as this review was conducted using an online database. Unlike systematic reviews, scoping reviews are not registered in the International Prospective Register of Systematic Reviews (PROSPERO).

Study Selection and Search Strategy

A systematic search of the English literature was conducted in the PubMed, Scopus, and Web of Science databases for articles published between January 1, 2012 and December 31, 2022, using Medical Subject Heading Terms (MeSH terms), as shown in Table 1.

**Table 1:** Search query for database

Database	Query	Results
PubMed	(radiosurgery OR gamma knife surgery OR stereotactic radiosurgery) AND (repeat OR second OR third OR retreatment) AND (trigeminal neuralgia OR tic douloureux) AND (recurrent OR refractory)	95
Scopus	All (radiosurgery OR gamma knife surgery OR stereotactic radiosurgery) AND (repeat OR second OR third or retreatment) AND (trigeminal neuralgia OR tic douloureux) AND (recurrent OR refractory)	58
Web of Science	All (radiosurgery OR gamma knife surgery OR stereotactic radiosurgery) AND (repeat OR second OR third or retreatment) AND (trigeminal neuralgia OR tic douloureux) AND (recurrent OR refractory)	42

**Figure 1:**
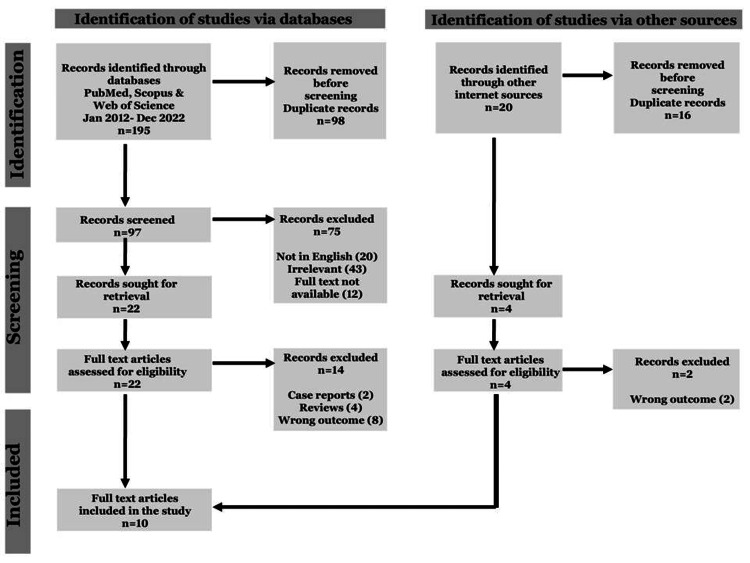
Prisma ScR flowchart PRISMA: Preferred Reporting Items for Systematic Reviews and Meta-Analyses

The PRISMA-ScR flow diagram of the data search is shown in Figure 1.

The first author (MG) conducted a search of the databases using MeSH terms from January 2012 to December 2022, as mentioned in Table 1. After eliminating duplicates, two independent reviewers (NP and JJ) screened the titles and abstracts of the relevant studies according to the eligibility criteria. After excluding non-relevant articles, the full texts of the selected articles were further screened to remove studies that did not meet the inclusion criteria. The reasons for the exclusion of articles were recorded and reported in this review. The reference lists of the included articles were searched for relevant studies. Any disagreements between reviewers at each stage of the selection process were resolved through discussion until a consensus was reached.

The patient/population, intervention, comparison and outcomes (PICOS) criteria for the search were as follows: P (population/patients), i.e., studies involving patients of any sex, and age, with recurrence of TD; I (intervention), i.e., using stereotactic radiosurgical procedure for treatment; C (comparator), i.e., comparison of the effectiveness of the SRS procedure, with initial treatments; O (outcome), i.e., the primary outcome was pain relief and the secondary outcome was post-SRS trigeminal dysfunction or facial numbness in the treatment of recurrent TD.

Eligibility Criteria for the Search

Inclusion criteria: All retrospective and prospective cohort studies, randomized control trials, studies between January 1, 2012 and December 31, 2022, studies where SRS has been used for initial and repeat treatments for recurrent type 1 TD cases, and primary studies in English were included.

Exclusion criteria: Case series, conference papers, theses, letters to the editor, editorials, case reports or series, animal studies, systematic reviews, meta-analyses, studies published before January 2011 and after December 2022, studies conducted on TD or atypical facial pain arising from tumor or other intracranial lesions (type 2 TD), studies conducted on post-mortem data or non-humans, and studies published in any language other than English were excluded.

Data Extraction

Data were extracted according to the PICOS criteria. The information on authors’ name, year, and type of publication; details of demographic data, such as age, sex, number of subjects, type of initial method used for assessment, rate of follow-up after the initial and final treatments, methods used for the initial treatment, dose of SRS for the management of recurrent TD; and outcome of the intervention were extracted from the studies.

Results

The initial database search resulted in 195 articles, and 20 additional records were identified through other sources. There was a perfect agreement between the two reviewers (kappa value=0.95) in the initial screening of titles and abstracts. Disagreements were resolved by a third reviewer. A total of 26 full-text articles were assessed for their eligibility. Ten studies were finally included [7,10,11,12,13,14,15,16,17,18] after excluding 12 studies, as shown in the PRISMA-ScR flow diagram (Figure 1). Seven studies used stereotactic radiosurgery for the second time [7,10,11,12,13,14,15], and three studies used SRS for the third time [16,17,18] for the management of recurrent or refractory TD. The details of these studies are presented in Tables 2-7.

**Table 2:** Demographic data of patients with second SRS procedure SRS: stereotactic radiosurgery

Author, year	No./total patients	Type of study	Male/female	Center	Age at second SRS (years)	Follow-up period (months)	Mean interval between the first and second SRS (months)
Park KJ et al., 2012 [10]	119/503	Retrospective	46/73	Pittsburgh, USA	74 (34–96)	48 (6–187)	26 (4–146)
Tuleasca C et al., 2014 [7]	13/737	Retrospective	Not categorized	Marseille, France	64.4 (53.7–83.1)	33.9 (15.9–96)	72 (12–125)
Hellis CA et al., 2015 [11]	152	Retrospective	63/89	North Carolina	68 (59-73)	27.1 (11.2-65.3)	18 (0-43)
Park SC et al.,2016 [12]	14/283	Retrospective	5/9	Seoul, Korea	65 (28-78)	11 (1-151)	45 (10-124)
Wang Yi et al., 2017 [13]	42/658	Retrospective	307/351	West China	54.3 (27-81)	74 (13-120)	34 (3-76)
Raygor KP et al., 2019 [14]	15/168	Retrospective	6/8	California	71 (55-85)	48 (3-93)	38 (4-72)
Marquez BS et al., 2022 [15]	202	Retrospective	55/147	South Miami, USA	70 (59-76.75)	12 (10.5-36)	15 (6-33)

**Table 3:** Details of dose and target area of irradiation for patients undergoing second SRS procedure RGZ: retrogasserian zone; Gy: gray; SRS: stereotactic radiosurgery

Author, year	Initial dose in Gy (range)	Second dose in Gy (range)	Cumulative dose in Gy (range)	Initial target	Second target
Park KJ et al., 2012 [10]	80 (60–90)	70 (50–90)	145 (120–170)	RGZ (100%)	50% overlap
Tuleasca C et al., 2014 [7]	85 (70–90)	90 (70–90)	175(140–180)	RGZ (100%)	Same as initial
Hellis CA et al., 2015 [11]	90 (85-90)	80 (80-85)	170 (165-175)	RGZ (100%)	Distally to the initial point
Park SC et al., 2016 [12]	85 (70-90)	85(50-90)	170 (140-180)	RGZ (100%)	Same as initial
Wang Yi et al., 2017 [13]	85 (70-90)	85 (70-90)	170 (140-180)	RGZ (100%)	Same as initial
Raygor KP et al., 2019 [14]	80 (60-85)	50 (40-80)	150 (140-155)	RGZ (100%)	Same as initial
Marquez BS et al., 2022 [15]	38 (38-39)	25 (25-26)	63 (63-64)	RGZ (50%)	50% overlap

**Table 4:** Outcome measures of the second SRS procedure BNI: Barrow Neurological Institute; SRS: stereotactic radiosurgery

Author, year	BNI class I–IIIb	BNI class I and II	No relief	Recurrence rate	Time of initial response	Trigeminal dysfunction	New facial numbness
Park KJ et al., 2012 [10]	87%	32%	13%	35% recurrence at 2 years	1.8 months (1-6)	18%	18%
Tuleasca C et al., 2014 [7]	89%	89%	11%	22.2% at 1 year	6 days (1-15)	33%	33.30%
Hellis CA et al., 2015 [11]	84%	46%	15%	20% after 5 years	1-3 weeks	5%	30% had mild facial numbness, 7% mild corneal dryness
Park SC et al., 2016 [12]	85.70%	Not mentioned	11%	Not mentioned	Not mentioned	11%	Not mentioned
Wang Yi et al., 2017 [13]	90.54%	33%	10%	41.12% at 5 years	14 days	23%	Not mentioned
Raygor KP et al., 2019 [14]	100%	76.90%	0%	0%	9.9 months (2.3-10)	40%	0%
Marquez BS et al., 2022 [15]	80%	3%	20%	40% at 1 year	4 months (1-9)	Not mentioned	18%

**Table 5:** Demographic data of the patients with a third SRS procedure SRS: stereotactic radiosurgery

Author, year	No./total patients	Type of study	Male/female	Age at the third SRS (years)	Follow-up period (months)	Mean interval b/w the first and second SRS (months)	Mean interval b/w the second and third SRS (months)
Tempel ZJ et al., 2015 [16]	17	Retrospective	7M/10F	79.6 (51.2-95.6)	22.9 (3-60)	35.2 (20-56)	38.1 (20-55)
Helis CA et al., 2020 [17]	22/152	Retrospective	10M/12F	74.7 (65.1-81.3)	24 (35-68)	24 (10-58)	60 (28-86)
Gupta M et al., 2021 [18]	30	Retrospective	14/16	69 (42-86)	39 (0.3-108)	33 (4-164)	33 (5-138)

**Table 6:** Details of the dose and target area of irradiation for patients undergoing a third SRS procedure RGZ: retrogasserian zone; Gy: gray; SRS: stereotactic radiosurgery

Author, year	Dose at the first SRS in Gy (range)	Dose at the second SRS in Gy (range)	Dose at the third SRS in Gy (range)	Cumulative dose in Gy (range)	Initial target	Second target	Third target
Tempel ZJ et al., 2015 [16]	80 (70-85)	70 (40-80)	70 (40-80)	210 (150-240)	RGZ (50%)	50% overlap anterior to the first SRS	Distal to the second SRS
Helis CA et al., 2020 [17]	90 (85-90)	80 (80-85)	75 (75-80)	245 (230-270)	RGZ (50%)	Distal to the first SRS	Proximal in 7 patients and distal in 15
Gupta M et al., 2021 [18]	81 (80-95)	81 (70-90)	81 (65-85)	243 (221-270)	RGZ (50%)	Same as the first SRS	Same as the first SRS but 20%

**Table 7:** Outcome measures of patients undergoing a third SRS procedure BNI: Barrow Neurological Institute; SRS: stereotactic radiosurgery

Authors, year	BNI class I–II after the first SRS	BNI class I-II after the second SRS	BNI class I-II after the third SRS	Recurrence rate	Mean time of recurrence (months)	Time of initial response (months)	Trigeminal dysfunction	New facial numbness
Tempel ZJ et al., 2015 [16]	35.30%	47.10%	47.10%	23.5% in 2 years	27 (19.1-34.8)	1 (1-4)	17.6% after the first, 11.8% after the second, 0% after the third SRS	Not mentioned
Helis CA et al., 2020 [17]	Not mentioned	46%	35%	18.2% in 3.9 years	46 (30-58)	0.7 (0-1.2)	0% after the third	10% new facial numbness after the third SRS, corneal dryness in 13.6% after the third SRS
Gupta M et al., 2021 [18]	23%	26%	29%	23% after 3 years	18 (4-76)	0.6 (1-2)	7 % after the first, 5% after the second, 8% after the third SRS	25% after the first, 18% after the second, 14% after the third SRS

Characteristics of the Included Studies

All included studies were retrospective and were published in medical neurosurgery journals. The age range of the samples in the studies was 30-80 years, and both males and females were included in the studies, except for one study, in which the sample was not categorized [7]. TN was more prevalent in females than in males, and on the right side, compared to the left or bilateral side, in all the included studies. The results revealed that seven studies were conducted in the USA [10,11,14,15,16,17,18], one in Korea [12], one in China [13], and one in France [7]. The mean follow-up period after the SRS procedure ranged from 11 to 50 months, and the mean interval between the first and second SRS procedures ranged from 15 to 40 months. Only one study had an extended time period of 72 months [7], whereas the time interval between the second and third SRS procedures ranged from 33 to 60 months [16,17,18]. A total of 515 patients underwent repeat second SRS procedure, and 69 patients underwent repeat third SRS procedure. All the studies reported pain predominantly in the ophthalmic (V2) and maxillary (V3) branches of the trigeminal nerve.

Dose of Irradiation

All therapeutic procedures were performed using the Leksell Gamma Knife (Elekta Instruments AB, Sweden). The emission of radiation was administered toward a solitary isocenter, facilitated through the utilization of a 4 mm collimator. The placement of the isocenter was observed to be along the trigeminal nerve, which is dysfunctional, usually at the entrance into Meckel’s cave or in the prepontine cistern. All the studies used almost the same radiation dose for the initial and repeat SRS procedures, in the range of 70-90 Gy, except one study that used a low radiation dose in the range of 25-40 Gy [15]. The radiation dosage for the initial and repeat GKS was almost identical in four studies [7,12,13,14,18], whereas it was shifted distally or proximally in other studies [10,11,15,16,17]. The radiation dose was delivered at the 100% isodose line, with the isocenter positioned at the retrogasserian zone (REZ), which is posterior to the gasserian ganglion, and the target placement varied to 8 mm, depending on the length of the nerve and its exit from the brainstem, except for four studies that used the 50% isodose line [15,16,17,18]. Gupta et al. used 20% isodose in the third SRS procedure. The cumulative dose for the two treatments ranged from 120 to 180 Gy, and for the three treatments, it ranged from 150 to 270 Gy. Only one study used a low cumulative dose for the repeat second GKS procedure of 63-64 Gy [15].

Treatment Outcomes

Pain response: Patient outcomes were evaluated using the Barrow Neurological Institute (BNI) pain scale [19]. The classification system consists of six distinct classes. In Class I, there is no trigeminal pain, and medication is not required. In Class II, there is occasional pain, which does not necessitate medication. In Class IIIa, patients do not experience pain with continuous medication. In Class IIIb, pain is managed with medication. In Class IV, there is some pain, but it is not effectively controlled with medication. Lastly, in Class V, patients experience severe pain without any relief. Successful treatment was denoted by complete pain relief (BNI score of I) or adequate pain relief (BNI score ranging from II to IIIb), whereas a score of IV or V was indicative of treatment failure, and patients underwent repeat SRS procedure. TN type 1 (TN1) is characterized by acute, piercing, electric shock-like, intermittent pain, which occurs more than 50% of the time, although it is interspersed with painless periods between episodes [1]. All studies included patients with TN1. Most patients with repeat SRS had more than 50% pain relief (BNI I-IIIB), in 80-90% of patients. One study reported 100% pain relief after repeat SRS [14]. Excellent pain relief (BNI I-II) was noticed in 30-50% of patients [10,11,13], 85-90% of patients in two studies [7,12,13], and only 3% of patients in one study [15]. In patients where three SRS procedures were performed, excellent pain relief in 25-35% during the first SRS and in 26-47% of patients in the second and third SRS procedures [16,17,18]. After the initial and subsequent SRS, a significant proportion of patients achieved pain relief within the first six months. The cessation rates of pain were consistent with the outcomes observed after the first SRS. A proportion (20-40%) of patients experienced pain recurrence after one year. Only one study reported no recurrence after SRS [14].

Toxicity: New paresthesia after a repeat SRS procedure was noticed in 18-33% of patients. After the third SRS procedure, none of the patients developed paresthesia in two studies [6,17], whereas only 8% of the patients developed paresthesia in one study [18]. Other complications include mild corneal dryness in 7-13.6% of patients [7,17] and mild facial numbness in 18-30% of patients [7,10,11,15,17,18].

Discussion

Although SRS is an effective procedure for the management of pain in TD, 20-40% of patients suffer from recurrent pain, for which the second and third SRS procedures are being undertaken. Several studies have attempted to establish a correlation between the response to SRS retreatment of recurrent TD and various factors, including but not limited to age, sex, duration of symptoms, comorbidities, type of trigeminal pain, prior surgical treatment, time between SRS treatments, SRS dose and number of isocenters, treated nerve length, relationship between the nerve root entry zone and the isocenter, and response to the initial SRS treatment.

Age, Gender, and Dose

Age, sex, and duration of symptoms prior to initial SRS were found to have no statistically significant impact on outcomes. Most of the studies have used 70-90 Gy radiation doses and cumulative dose of 120-180 Gy for two SRS procedures and 150-270 Gy for three SRS procedures. Park et al. reported that initial doses less than 60 Gy and cumulative doses less than 150 Gy were less effective in pain control [12]. According to Drovak et al., patients with debilitating TD should receive cumulative doses of >150 Gy, and patients with BNI II scores, who suffer from side effects of medications, should receive a lower dose [20]. Increased radiation doses have been found to have better pain control but are also associated with trigeminal dysfunction. Marquez et al. used a low radiation dose of 25-30 Gy, and only 3% patients had BNI I-II scores [15].

Target

All studies used a retrogasserian target for radiation. The use of a retrogasserian target confers a substantial advantage in minimizing the radiation dose to the brainstem, potentially mitigating complications that may arise from trigeminal sensory- and brainstem-related factors. It is recommended that brainstem doses be maintained below 12 Gy by utilizing target distances that exceed 5 mm from the surface of the brainstem to minimize side effects. Repeat SRS procedures with cumulative brainstem doses exceeding 12 Gy have been associated with postoperative toxicity, and 10 Gy has been suggested as a threshold for cranial nerve nuclei inside the brainstem [12]. Only one study mentioned brainstem dosage, which was kept below 12 Gy. Radiation effects on the brainstem can be minimized by using the 50% isodose line, which has been used in previous studies [15,16,17,18]. It can also be reduced by shifting the irradiation position either distally or proximally in a repeat SRS procedure, as has been done in many studies [10,11,15,16,17]. Increasing the distance from the brainstem to more than 5 mm can also help reduce the effects of radiation [7,11,16].

Outcome After Repeat SRS

Consideration of the radiation target is of significant importance in relation to the initial cessation of pain and its potential side effects. The diverse range of radiation targets highlighted in existing publications creates challenges in establishing "optimal" targets. All studies reported pain relief (BNI I-IIIb) in 80-90% of cases within four months of treatment. A proportion of 30-40% patients reported excellent pain relief (BNI I). The pain cessation rates after repeat SRS were consistent with those after the first SRS. Raygor et al. reported pain relief in 100% patients, with excellent pain relief in 76% patients [14]. The reason for the difference in pain response could be the small sample size of 15 patients. Pain recurrence was noticed in 20-40% of cases after one year. The probable reason for the recurrence might be due to the fact that the impact of whole-brain radiation on the white matter is predominantly recognized as a demyelinating effect that is dependent on the dosage administered. This results in a reduction in fractional anisotropy and an increase in the mean diffusivity in the diffusion tensor images. The progression of this effect on the white matter was observed for up to one year following treatment, as reported in the literature. However, after the one-year mark, it is common for TN to reoccur, and the biological influence of SRS diminishes gradually [12]. Positive predictive factors for pain cessation rates taken from the included studies were post-sensory changes after the first SRS [14], facial numbness after the first SRS [17], complete pain relief after the initial SRS [17], and longer periods of pain relief [12]. Negative predictors are no pain relief after prior SRS [17] or long inter-treatment intervals [12]. No correlation was found between age, sex, time of pain recurrence, TN distribution, additive doses, or interval between the first and second SRS [14].

Toxicity

It has been documented in the scientific literature that the likelihood of exhibiting symptoms associated with trigeminal nerve dysfunction is elevated subsequent to undergoing retreatment with SRS. New facial sensory symptoms caused by partial trigeminal nerve injury are the primary complications observed after repeat SRS. These symptoms are seen in 18-33% of patients. Other complications include corneal dryness, which can be mild to bothersome, alteration in taste sensation, jaw weakness, and anesthesia dolorosa [20]. The reason for these sensory disturbances could be the high radiation dose used for pain control in type 1 TD cases. The role of brainstem edge dose and trigeminal nerve deficits is controversial [12]. The inconclusive findings regarding brainstem doses and their correlation with trigeminal nerve deficits may suggest that the brainstem dose by itself is not the sole determinant of trigeminal nerve deficits. Trigeminal nerve deficits are commonly associated with pain control outcomes. Therefore, it is our conjecture that high-dose radiation leads to the disconnection of the trigeminal tract, as demonstrated in a recent diffusion tensor imaging study, and brainstem radiation can potentially result in trigeminal nerve deficits. Complications can be minimized by reducing the brainstem dose and changing the target location for repeat SRS, as has been done in previous studies [10,11,15,16,17].

The predictive factors for new hypesthesia after the second SRS are isocenter placement (distally placed shots were associated with fewer complications compared to proximally placed shots), distance of isocenter from pons, cumulative radiation dose to pons, and cumulative radiation dose to REZ [16]. No significant correlation was observed among prior surgical procedures, sensorial deficits prior to treatment, and spatial separation between isocenters [7].

Limitations

Due to the heterogeneity in the studies regarding the optimal dose and optimal target zone for the management of recurrent TD, the present review failed to address the two important research questions. All included studies were retrospective and did not mention the brainstem edge dose, which is a very important factor regarding post-SRS complications. Multicenter prospective studies with a long follow-up period are required in the future.

## Conclusions

Studies have indicated that the rates of pain control and recurrence after repeat SRS are similar to those of initial SRS. SRS is a relatively safe and noninvasive procedure that can be effectively used for pain management in type 1 TD. To scrutinize the safety and efficacy of this procedure, it is imperative to meticulously consider the individual patient characteristics at baseline and the technical intricacies, particularly the placement of the target and the amount of radiation dosage administered. Furthermore, it is crucial to consider the number of patients involved and the duration of follow-up, as these are important criteria for the evaluation of this treatment.
